# In-Plane Resonant Nano-Electro-Mechanical Sensors: A Comprehensive Study on Design, Fabrication and Characterization Challenges

**DOI:** 10.3390/s130709364

**Published:** 2013-07-22

**Authors:** Faezeh Arab Hassani, Yoshishige Tsuchiya, Hiroshi Mizuta

**Affiliations:** 1 School of Materials Science, Japan Advanced Institute of Science and Technology (JAIST), Nomi, Ishikawa 923-1292, Japan; E-Mail: mizuta@jaist.ac.jp; 2 School of Electronics and Computer Science, University of Southampton, Southampton SO17 1BJ, UK; E-Mails: yt2@ecs.soton.ac.uk (Y.T.); hm2@ecs.soton.ac.uk (H.M.)

**Keywords:** nano-electro-mechanical sensors, resonance detection, total quality factor, junction-less field-effect-transistor, metal-oxide-semiconductor field-effect-transistor

## Abstract

The newly proposed in-plane resonant nano-electro-mechanical (IP R-NEM) sensor, that includes a doubly clamped suspended beam and two side electrodes, achieved a mass sensitivity of less than zepto g/Hz based on analytical and numerical analyses. The high frequency characterization and numerical/analytical studies of the fabricated sensor show that the high vacuum measurement environment will ease the resonance detection using the capacitance detection technique if only the thermoelsatic damping plays a dominant role for the total quality factor of the sensor. The usage of the intrinsic junction-less field-effect-transistor (JL FET) for the resonance detection of the sensor provides a more practical detection method for this sensor. As the second proposed sensor, the introduction of the monolithically integrated in-plane MOSFET with the suspended beam provides another solution for the ease of resonance frequency detection with similar operation to the junction-less transistor in the IP R-NEM sensor. The challenging fabrication technology for the in-plane resonant suspended gate field-effect-transistor (IP RSG-FET) sensor results in some post processing and simulation steps to fully explore and improve the direct current (DC) characteristics of the sensor for the consequent high frequency measurement. The results of modeling and characterization in this research provide a realistic guideline for these potential ultra-sensitive NEM sensors.

## 1. Introduction

The co-integration of micro/nano-electrical and micro/nano-mechanical devices is expected to lead to the development of future smart sensors [1]. Monolithically integrated micro/nano-electro-mechanical systems (MEMS/NEMS) and integrated circuits (ICs) even push smart sensors towards more advanced applications taking advantage of the benefits of both technologies [2,3]. Among different existing sensing methods for NEM sensors, the mass detection based sensors are very popular due to the higher resolution and accuracy of frequency measurement in response to very small changes in mass [4,5]. The usage of monolithically integrated metal-oxide-semiconductor field-effect-transistor (MOSFET) with NEM sensors eases the resonance detection of these sensors due to the shorter physical distance between the NEM sensor and transistor [6–8].

In this paper, first we propose an in-plane resonant nano-electro-mechanical (IP R-NEM) sensor based on silicon-on-insulator (SOI) technology. The uniform doping of the suspended beam and side electrodes of this sensor provide the opportunity of realizing a FET with no junctions and doping concentration gradients, a so-called junction-less field-effect-transistor (JL FET), with two side gates. Then, the suspended beam along with one side electrode is integrated with an in-plane MOSFET to realize the second NEM sensor named as in-plane resonant suspended gate FET (IP RSG-FET) sensor. In Section 2, we present the design, structure, analytical and numerical key parameters of both sensors. The modeling of the sensing process that consists of the functionalization and detection processes are discussed in Section 3, followed by the calculation of mass responsivity using analytical and numerical techniques. Section 4 presents the fabricated sensors followed by their fabrication processes. Finally, the direct current (DC) characteristics of the sensors are conducted for the consecutive high frequency characterization of the sensors in Section 5.

## 2. Design and Structure of Nano-Electro-Mechanical (NEM) Sensors

**Figure 1.**
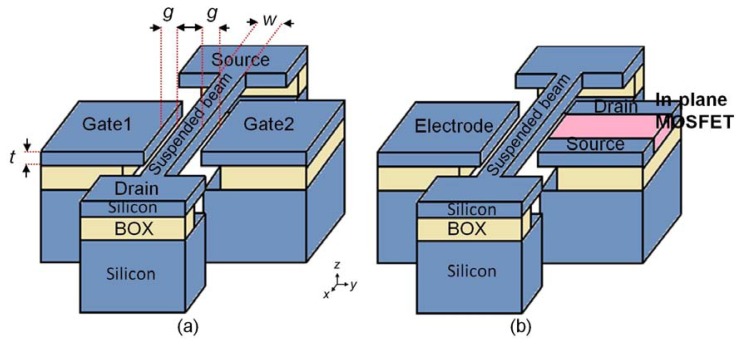
**(a)** The IP R-NEM sensor: A suspended beam that acts as the channel for the junction-less field-effect-transistor (JL FET) with two side gates. (**b**) The in-plane resonant suspended gate field-effect-transistor (IP RSG-FET) sensor: A suspended beam that is integrated with an in-plane metal-oxide-semiconductor FET (MOSFET).

The IP R-NEM sensor consists of a suspended clamped-clamped (CC) beam and two side electrodes (Figure 1a). The sensor is fabricated based on SOI technology considering the uniform doping for the whole structure. The suspended beam is later considered as the laterally resonating channel for the JL FET. The CC beam is excited by an alternating current (AC) voltage due to the equality of its resonant frequency with the frequency of the AC voltage. The changes in the displacement of the CC beam cause changes in the current of the JL FET that is used for the detection of the resonance frequency. This is one possible detection method for the sensor besides the capacitance detection method. For the second sensor, an in-plane MOSFET is integrated with the suspended beam, IP RSG-FET sensor (Figure 1b). In this structure, the beam acts as the suspended gate for the MOSFET that moves laterally and induces current in the channel of the MOSFET. In both sensors, the adsorbed linker and target molecules on the surface of the beam changes its resonance frequency and as a result causes variation in the current of JL FET or MOSFET.

### 2.1. Analytical Calculations

The resonance frequency of the first lateral mode of the clamped-clamped beam is calculated by [9–11]:

(1) $$f_{0}=1.03\sqrt{\frac{E}{\rho }}\frac{w}{l^{2}}=\frac{1}{2\pi }\sqrt{\frac{k_{bm}}{m_{b}}}$$

where *E*, is Young's modulus, *ρ*, density, and, *w*, and, *l*, the width and length of the beam, respectively, *m_b_*, the effective mass of the beam, *k_bm_*, the mechanical spring stiffness of the beam. *m_b_* and *k_bm_* are given by [12]:

(2) $$m_{b}=0.735\rho \text{wlt}$$

(3) $$k_{bm}=30.78Et{\left(\frac{w}{l}\right)}^{3}$$

where *t*, is the thickness of the beam. The effective spring stiffness, *k_b_*, is defined by:

(4) $$k_{b}=k_{bm}+k_{be}$$

where *k_be_* is the electrical spring stiffness and calculated by [13–15]:

(5) $$k_{be}=\frac{-\varepsilon_{0}AV_{dc}^{2}}{g^{3}}$$

where the air permittivity, *ε_0_*, is 8.85 × 10^−12^ F/m, *g* is the gap between the beam and electrodes, the beam area, *A*, is equal to *l* × *t* and *V_dc_* is the applied DC voltage to the beam. As a result, the resonance frequency is calculated as follows:

(6) $$f_{r}=\frac{1}{2\pi }\sqrt{\frac{k_{b}}{m_{b}}}$$

In order to operate the NEM sensor with a safe margin, *V_dc_* will be chosen to be well below the pull-in voltage and assumed that *k_b_* ≈ *k_bm_* and consequently *f_r_* ≈ *f_0_*.

Energy is dissipated from the resonator by different damping mechanisms. In general, the energy in the resonator is dissipated via the ambient, *Q_Ambient_* (gas/liquid damping), through the anchors, *Q_Anchor_* (anchor damping/loss), and its material itself, *Q_Thermoelastic_* (thermoelastic damping) [16]. *Q_Total_* is defined as the ratio of the total energy stored in the system to the energy dissipated or lost per cycle of vibration and is calculated as follows [16]:

(7) $$\frac{1}{Q_{\text{Total}}}=\frac{1}{Q_{\text{Ambient}}}+\frac{1}{Q_{\text{Anchor}}}+\frac{1}{Q_{\text{Thermoelastic}}}$$

*Q_Ambient_*, is calculated by [17]:

(8) $$Q_{\text{Ambient}}=\frac{\sqrt{k_{b}m_{b}}}{b}$$

The damping factor, *b*, is calculated as follows [18,19]:

(9) $$b=\frac{96}{\pi^{4}}\frac{\mu lt^{3}}{g^{3}}$$

where *μ* is the viscosity of the medium around the beam. For nano-scale resonators with the gap smaller than the air mean-free path, *λ_atm_*, of 68 nm in the atmosphere pressure, *P_atm_*, *μ* is not independent of pressure [20,21], and is defined using the Reynolds equation [22]:

(10) $$\mu =\frac{\mu_{0}}{1+9.638K_{n}^{1.159}}$$

where *μ_0_* is the air viscosity and *K_n_* is the Knudsen number that is calculated by [19]:

(11) $$K_{n}=\frac{\lambda_{0}}{g}=\frac{P_{n}\lambda_{n}}{P_{0}g}$$

where *λ_0_* is the air mean-free path at the operating pressure and *λ_n_* is the air mean-free path at a known pressure, *P_n_*.

The inverse quality factor due to the thermoelastic damping, $Q_{\text{Thermoelastic}}^{-1}$, for dimensions down to nano-scale and the temperature above 100 K is calculated by [23]:

(12) $$Q_{\text{Thermoelastic}}^{-1}=\frac{E\alpha^{\prime 2}T}{C}\left(\frac{6}{\xi^{2}}-\frac{6}{\xi^{3}}\frac{\sinh\xi +\sin\xi }{\cosh\xi +\cos\xi }\right)$$

where *α′* is the thermal expansion, *T* is the operating temperature and *C* is the heat capacity of the beam. *ξ* is defined by [23]:

(13) $$\xi =w\sqrt{\frac{2\pi f_{0}}{2\chi }}$$

where *χ* is the solid's thermal diffusivity.

The anchor damping for nano-scale resonators in [24] is defined due to the tunneling of phonons between the beam and its anchors, which is calculated by [24]:

(14) $$Q_{\text{Anchor}-\text{phonon}}=\frac{3.9l^{5}}{2\pi^{4}\tanh^{2}\frac{1.5\pi }{2}tw^{4}}$$

However, the anchor damping, *Q_Anchor_*, in [25] is derived based on a two dimensional elastic theory as follows:

(15) $$Q_{\text{Anchor}}=\frac{2\times 2.43}{(3-\vartheta )(1+\vartheta ){\left(\beta_{n}X_{n}\right)}^{2}}{\left(\frac{l}{w}\right)}^{3}$$

where *ϑ* is the Poisson's ratio, *X_n_* is the shape factor and *β_n_* is the mode constant for a CC beam and *n* shows the mode number of the resonator. An equivalent circuit model is considered for the sensor that consists of a capacitor, an inductor and a resistor, *R_x_* [26]. *R_x_* affects the magnitude of the output signal of the sensor and this effect becomes more important for nano-scale sensors [22]. *R_x_* is calculated as follows [26]:

(16) $$R_{x}=\frac{\alpha }{\eta^{2}}$$

where *α*, and *η* are calculated by:

(17) $$\alpha =\frac{2\pi f_{0}m_{b}}{Q_{\text{Total}}}$$

(18) $$\eta =\frac{V_{p}\varepsilon_{0}A}{g^{2}}$$

The parameters used for the calculation of *Q_Total_* and other key parameters such as resonant frequency, mass, and spring stiffness for the sensors are shown in Table 1. We assume sensors work at atmosphere and temperature of 300 K. *Q_Anchor-phonon_* was not considered for the calculation of *Q_Total_* for the sensor as it was a few orders of magnitude larger than other components of *Q_Total_* and did not limit the total quality factor. As shown in Table 1, first *Q_Ambient_* and then *Q_Anchor_* are smaller than *Q_Thermoelastic_* and restrict *Q_Total_*. By using the vacuum and low temperature for working condition of the resonator, *Q_Ambient_* and *Q_Thermoelastic_* will be reduced respectively and *Q_Anchor_* plays the dominant role among the damping sources. The anchor damping can be reduced to zero by properly designing the sensor using a free-free beam. The details of this concept will be presented elsewhere.

### 2.2. Numerical Analysis

**Figure 2.**
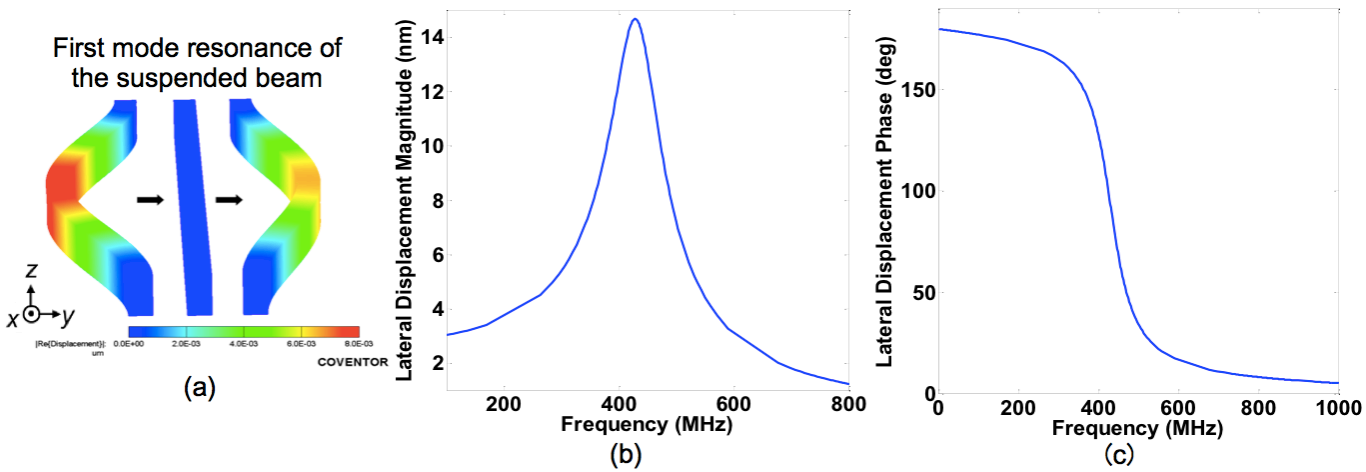
**(a)** The first resonance mode of the suspended beam. (**b**) The magnitude and (**c**) phase of in-plane displacement of the suspended beam *versus* frequency in the presence of a sinusoidal pressure load.

CoventorWare [27], was used for simulating the NEM sensors. This suite consists of a three dimensional finite-element-method (3D FEM) part including Designer and Analyzer and a circuit-level module, Architect. First, we conducted the 3D FEM analysis for the IP R-NEM sensor. Then, one of the electrodes in the IP R-NEM sensor was substituted with a lateral MOSFET in circuit-level modeling using Architect to realize the IP RSG-FET sensor. The first in-plane mode of the beam is obtained by using Analyzer at the frequency of 432.77 MHz as shown in Figure 2a. The magnitude and phase of the in-plane displacement *versus* frequency for the beam in the presence of a sinusoidal pressure load with the magnitude of 1 kPa are shown in Figure 2b,c, respectively. When the frequency of the applied pressure is equal to the resonance frequency of the beam, it resonates and shows a large peak for the displacement at the resonance frequency as shown in Figure 2b. Figure 2c shows also a change of 180 deg for the displacement of the beam at the resonance frequency. The derived numerical values including the resonance frequency, damping factor and quality factors are shown in Table 2, which are consistent with the analytical values in Table 1.

**Figure 3.**
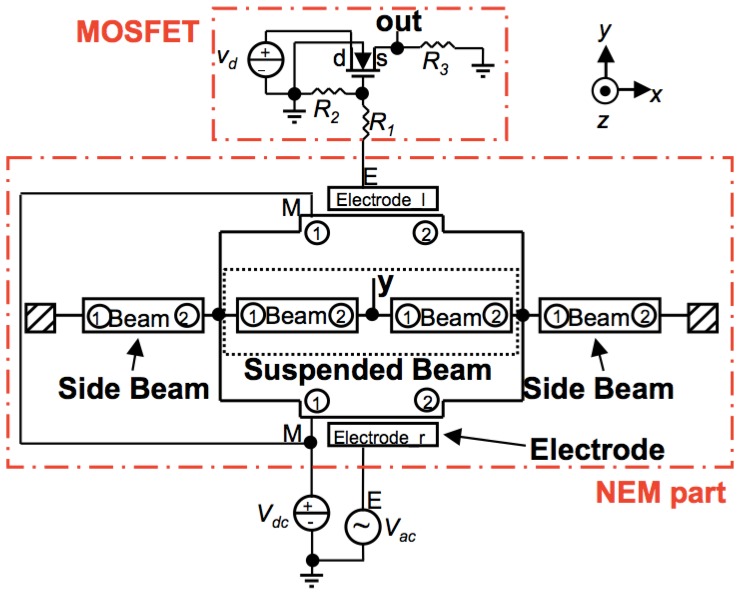
The hybrid circuit model of the in-plane resonant suspended gate filed-effect-transistor (IP RSG-FET) sensor in Architect.

Architect was used to prepare a hybrid NEM-MOS circuit model to analyze the IP RSG-FET sensor. The hybrid circuit model in Figure 3 consists of a NEM part (a suspended beam and side electrodes) and a MOSFET. The suspended beam is modeled using two central beam components, to model the node at the center, with two side beam components to avoid the existing limitations of the software. A DC voltage is applied to the beam and an AC voltage is applied to the side electrode for biasing. The node ‘out’ in Figure 3 denotes the output voltage of the sensor. The values for resistance, *R_1_* and *R_2_*, in this model were chosen with the smallest possible value to ensure the sensor resonance behavior remains valid. The n-type MOSFET has the 1 μm-channel length, which is the same as the length of the beam.

**Figure 4.**
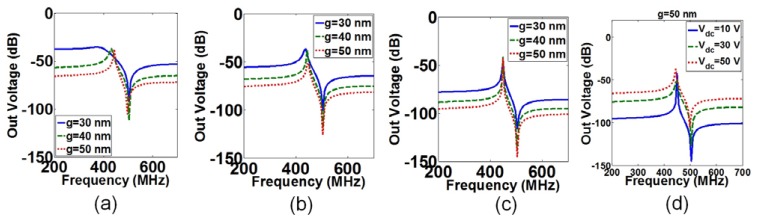
The out voltage of the in-plane resonant suspended gate field-effect-transistor (IP RSG-FET) sensor *versus* frequency at *V_d_* = 10 mV for different gaps at: (**a**) *V_dc_* = 50 V, (**b**) *V_dc_* = 30 V and (**c**) *V_dc_* = 10 V. (**d**) The out voltage of the IP RSG-FET sensor *versus* frequency for *g* = 50 nm at different *V_dc_*.

The gap was changed from 50 to 30 nm for three different DC voltages in order to investigate the effects of gap changes on the resonance frequency. The output voltage *versus* frequency for different gaps and voltages are shown in Figure 4, in which the left peaks present the resonance frequency while the right peaks denote the anti-resonance frequency [26]. Figure 4a shows that the resonance frequency shifts to lower frequencies by decreasing the gap at *V_dc_* = 50 V. This is due to the fact that by reducing the gap, *k_be_* in Equation (5) increases and results in smaller *k_b_* with respect to Equation (4) and consequently lower *f_r_* based on Equation (6). For *V_dc_* = 30 V in Figure 4b, this effect is only visible when the gap reduced from 40 nm to 30 nm. By reducing the gap at *V_dc_* = 10 V in Figure 4c, there will not be a shift for the resonance frequency as the effect of *k_be_* on *k_b_* is smaller for smaller *V_dc_*. Figure 4a–c shows that the widening of the resonance frequency spectrum is larger by reducing the gap in different voltages, which means a smaller *Q_Total_*. This fact is explained by the dominant effect of *Q_Ambient_* in these results, since it was assumed that the sensor works in the atmosphere and at room temperature. By reducing the gap with respect to Equation (9), the damping factor increases and results in smaller *Q_Ambient_* due to Equation (8). The output voltage *versus* the resonance frequency for *g* = 50 nm at *V_dc_* = 10 V to 50 V are shown in Figure 4d. Figure 4d shows that by increasing *V_dc_* from 10 V to 50 V, the resonance frequency decreased due to increasing of *k_be_* in Equation (5) and causes reduction in *k_b_* and consequently reduction in the resonance frequency with respect to Equation (4). The reduction of the resonance frequency with increasing of *V_dc_* is called spring softening [14]. Reduction in *k_b_* causes smaller *Q_Ambient_* and wider resonance frequency spectrum similar to Figure 4a–c.

## 3. Sensing Process of Nano-Electro-Mechanical (NEM) Sensors

The sensing process of NEM sensors consists of two steps of functionalization and detection processes. The selective detection of particular biological or chemical molecules are possible by the functionalization of the surface of the suspended beam using various self-assembled monolayer (SAM) linker molecules, such as Amino-propyltrimethoxysilane (APTES) as a silane coupler, or alkene/alkyne-based molecules.

### 3.1. Analytical Calculations

**Figure 5.**
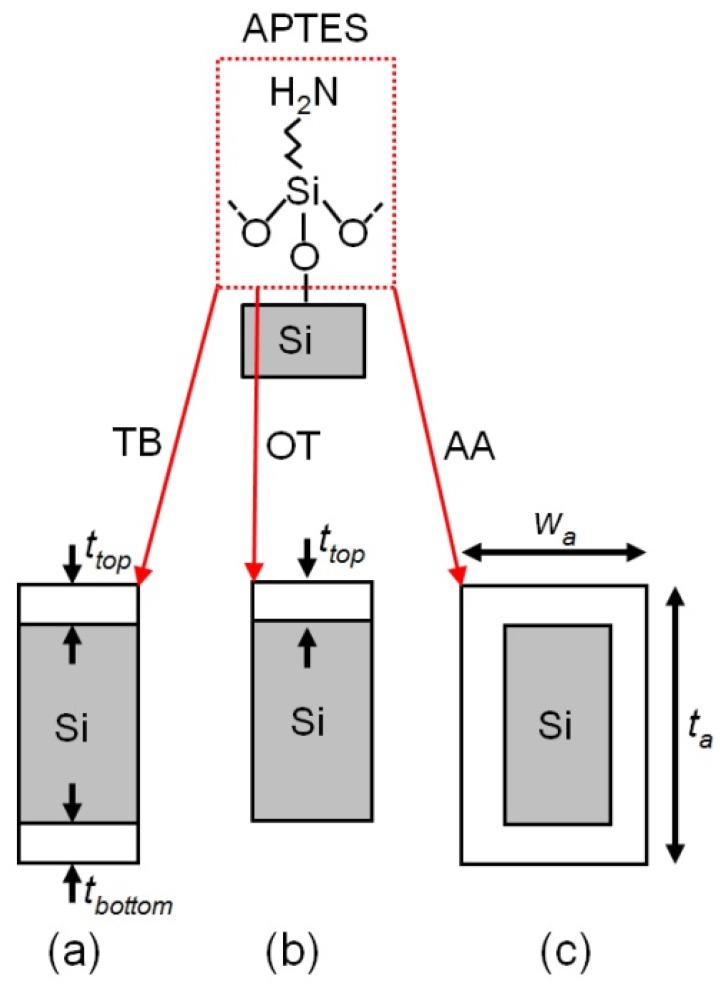
The schematic of the functionalized silicon surface with Amino-propyltrimethoxysilane (APTES) in different coating configurations: (**a**) top and bottom (TB), (**b**) only top (OT), and (**c**) all-around (AA).

Linker molecules are supposed to coat the surface homogeneously, for this reason, we have modeled these molecules simply by adding an extra surface coating layer onto the suspended beam. The surface functionalization processes are performed in reality either in liquid or in vapor that result in different surface coating configurations. We studied three coating configurations: top and bottom (TB), only top (OT) and all-around (AA) coating. For the TB configuration in Figure 5a two surface layers with the same thickness are considered on the top and bottom of the beam. This configuration is the suitable model to describe the coating in liquid as the molecular solution flows more freely above and under the beam rather than through the nano-scale gap between the suspended beam and the electrodes. We also considered the OT configuration (Figure 5b) for the case that the gap underneath the suspended beam is as small as the side gaps. The AA configuration shown in Figure 5c is also a likely case for the coating in vapor, as the vapor flow can go through narrow gaps more easily than the liquid flow. This configuration may also be applicable for the liquid phase if the side and bottom gaps are wide enough so the solution reaches all surfaces of the suspended beam easily. The total mechanical spring stiffness for the TB configuration, *k_b-TB_*, is given by [7]:

(19) $$k_{b-TB}=30.78{\left(w/l\right)}^{3}[Et+E_{c}(t_{\text{top}}+t_{\text{bottom}})]$$

where *E_c_*, is the young's modulus of the coating layer, *t_top_*, and, *t_bottom_*, the thickness of top and bottom layers (*t_top_* = *t_bottom_*). The calculated mass for the beam with the TB configuration is [7]:

(20) $$m_{b-TB}=m_{b}+0.735\rho_{c}w(t_{\text{top}}+t_{\text{bottom}})l$$

where *ρ_c_*, is the density of the coating layer. Equations (19) and (20) can be used for the OT configuration considering *t_bottom_* = 0. The total mechanical spring stiffness for the AA configuration, *k_b_*_–_*_AA_*, is calculated by [7]:

(21) $$k_{b-AA}=30.78\{Et{\left(w/l\right)}^{3}+[(E_{c}/l^{3})(t_{a}w_{a}^{3}-tw^{3})]\}$$

where *t_a_*, and, *w_a_*, are the thickness and width of the beam after adding the coating layer, respectively. The calculated mass for the beam with the AA configuration is [7]:

(22) $$m_{b-AA}=m_{b}+0.735\rho_{c}l(w_{a}t_{a}-wt)$$

Equations (19–22) show both spring stiffness and mass will increase by increasing the thickness of the coating layer and affects the resonance frequency. To find out the dominant factor between them, the changing rates of mass, (*Δm_bl_*/*m_b_*), and spring stiffness, (*Δk_bl_*/*k_b_*), were considered as representative parameters in Table 3. Here *Δm_bl_* and *Δk_bl_* are changes of the mass and spring stiffness due to the linker molecules and a 1 nm-thick coating layer is used as an example for the calculations. Table 3 shows that by increasing the thickness of the coating layer in both OT and TB configurations the increase of mass is higher than that of the spring stiffness while for the AA configuration the spring stiffness plays the dominant role. According to the dominant parameter for each configuration in Table 3, the changes in resonance frequency, *Δf_0_*, will be positive or negative by considering Equation (1). Note that, *Δf_0_* > *0* shows the increase in *f_0_* by increasing the thickness of the coating layer in the AA configuration, while *Δf_0_* < *0* presents the reduction of *f_0_* by increasing the thickness of the coating layer in the OT and TB configurations. This effect is also validated using the simulation later. Previous studies [28,29] on micro-resonators, show that the deposition of coating materials and adsorption of an analyte can affect not only the mass but also the spring stiffness. This effect results in a change in the resonance frequency towards higher or lower values.

In order to model the sparse and random adsorption of target molecules to the functionalized surface we simply increased the density of the coating layer with various configurations because the target molecules will change the effective mass of the beam without much affecting the spring stiffness of the beam. By using this method, we are able to study the impact of solely the mass increase due to the adsorbed target molecules on the resonant frequency and evaluate the mass responsivity of the sensor for the detection process. The numerical analysis for the mass responsivity is given in the next section. Using Equation (6), the mass responsivity, *S*, is calculated as follows [30]:

(23) $$S\approx \frac{\partial m_{b}}{\partial (2\pi f_{0})}=\frac{-m_{b}}{\pi f_{0}}$$

By using the values in Table 1, *S* is calculated and equal to 0.007 zepto g/Hz, which is then compared with the numerical value in the next section. Considering several noise processes in the operation of a resonator, the changes of the mass due to the adsorbed molecules, *Δm_ba_*, is calculated as follows [31]:

(24) $$\Delta m_{ba}\approx S{\left(\Delta f_{BW}\frac{2\pi f_{0}}{Q_{\text{Total}}}\right)}^{1/2}10^{\left(-DR/20\right)}$$

where *Δf_BW_* is the maximum allowable measurement bandwidth that is ∼*f_0_*/*Q_Total_* and *DR* represents the effective dynamic range intrinsic to the resonator [31]:

(25) $$DR(dB)=10\log(E_{C}/k_{B}T)$$

where Boltzmann constant, *k_B_*, is 1.38 × 10^−23^ J/K and the maximum drive energy for the in-plane CC beam is $E_{C}\approx m_{b}4\pi^{2}f_{0}^{2}0.53^{2}w^{2}$. Using Equations (23–25), *Δm_ba_* is calculated and equal to 1.6 zepto g. By using a high vacuum and low temperature environment for the sensor, *Δm_ba_* will reduce further.

### 3.2. Numerical Analysis

**Figure 6.**
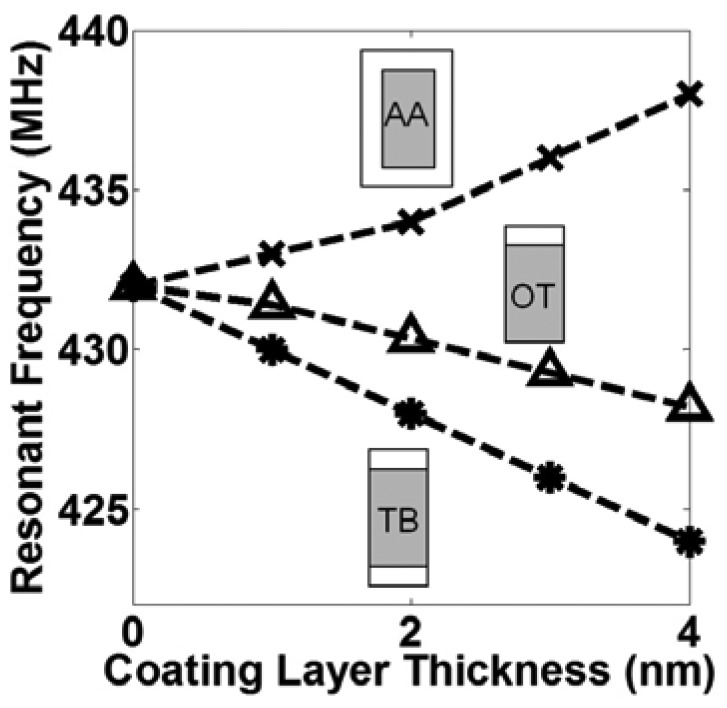
The resonant frequency *versus* coating layer thickness of the top and bottom (TB), only top (OT) and all-around (AA) configurations for the functionalization process in Designer.

Various insulator/polymer materials were used for modeling the functionalization process of the IP R-NEM sensor by adding a coating layer in different configurations in Designer to realize a homogenous and dense SAM layer in reality. We assumed for the present 3D FEM simulation that both ends of the coating layer were not anchored. This assumption may cause some differences from the results of the analytical model in Table 3, which assume the coating layer was also doubly clamped. In reality, it depends on the details of the surface coating of the resonator at the clamping points whether or not the non-anchored model is more appropriate. Figure 6 shows the resonance frequency *versus* the coating layer thickness for the TB, OT and AA configurations. In Figure 6, the frequency decreases in the TB and OT configurations by increasing the coating layer thickness. This trend shows the dominant effect of mass on the resonance frequency, which is consistent with the results in Table 3. Change in the resonance frequency is lower for the OT configuration than the TB configuration, due to its smaller mass value of the coating layer. Higher resonance frequencies for the OT configuration are explained by the smaller mass of the coating layer for the OT configuration than that of the TB configuration. On the contrary, the resonance frequency increases by increasing the coating layer thickness in Figure 6, as expected from Table 3 because of the dominant effect of the spring stiffness enhancement for the AA configuration.

**Figure 7.**
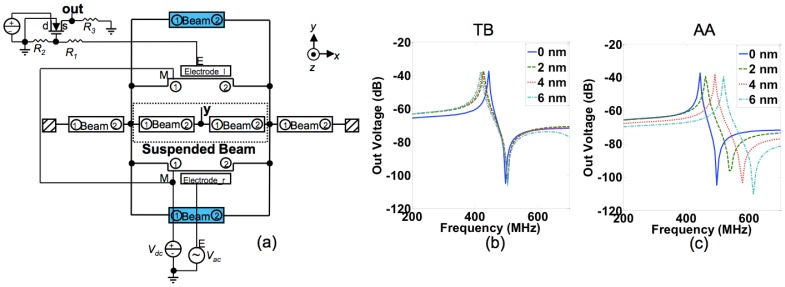
**(a)** The hybrid circuit model of the in-plane resonant suspended gate field-effect-transistor (IP RSG-FET) sensor with the added coating layers for modeling the top and bottom (TB) and all-around (AA) configurations in Architect. The output voltage of the IP RSG-FET sensor *versus* frequency for different thicknesses of the coating layer in Architect for: (**b**) the TB configuration and (**c**) the AA configuration.

To study the impact of adding the coating layer on the output voltage of the designed IP RSG-FET sensor, we introduced the coating layer onto the beam in our circuit model by adding extra beam components as shown in Figure 7a. The same assumption of non-anchored ends for the coating layer is also applied here. Figure 7b,c shows the output voltage *versus* frequency calculated with different coating layer thicknesses of the TB and AA configurations. The frequency spectra of the output voltage shift to low frequencies by increasing the thickness of the coating layer for the TB configuration (Figure 7b) while the trend is reverse for the AA configuration (Figure 7c). These results show the effect of the mass change is dominant for the TB configuration and the effect of the spring stiffness change is dominant for the AA configuration as explained previously in this section. These results are consistent with the previous 3D simulation results in Figure 6.

**Figure 8.**
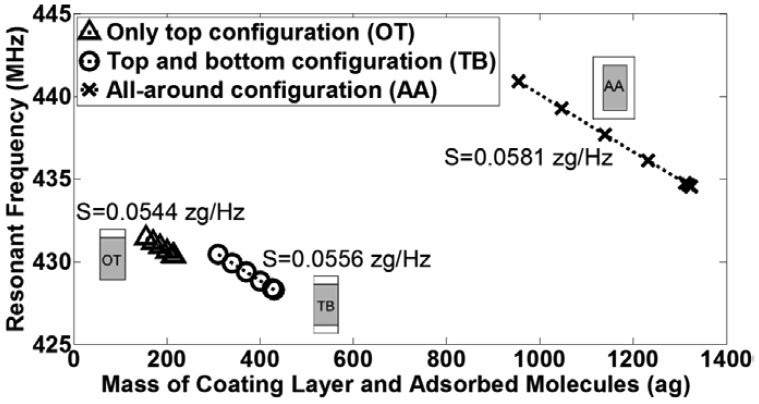
The resonant frequency *versus* mass of coating layer and adsorbed molecules of top and bottom (TB), only top (OT) and all-around (AA) configurations for the detection process in Designer.

The density of the added coating layer in different configurations for the functionalization process has been increased to model the adsorbed target molecules for the detection process. The resonant frequency for all coating configurations *versus* the total mass of the surface coating layer (2 nm-thick) and the adsorbed target molecules are shown in Figure 8. Regardless of the different coating schemes, the increase in the mass due to the adsorbed molecules decreases the resonant frequency linearly. The inverse slope of the resonant frequency *versus* mass shows the mass responsivity, *S*. Figure 8 shows virtually the same *S* values of 0.05 zeptogram/Hz for all configurations regardless of modified resonant frequency with different functionalization schemes and independent of the surface area of the beam that is used for adsorption. The calculated numerical value for *S* is one order of magnitude larger than the calculated analytical value in the previous section. This fact can be explained due to the assumption of non-anchored coating layer as well as the difference between the analytical and numerical values for the resonance frequency.

A few of the most recent research works on mass detection based NEM sensors using a CC beam with different materials [4,12,30,32,33], are given in Table 4. The proposed NEM sensors in this paper show higher sensitivity than that of the stated sensors in Table 4.

## 4. Fabrication of Nano-Electro-Mechanical (NEM) Sensors

**Figure 9.**
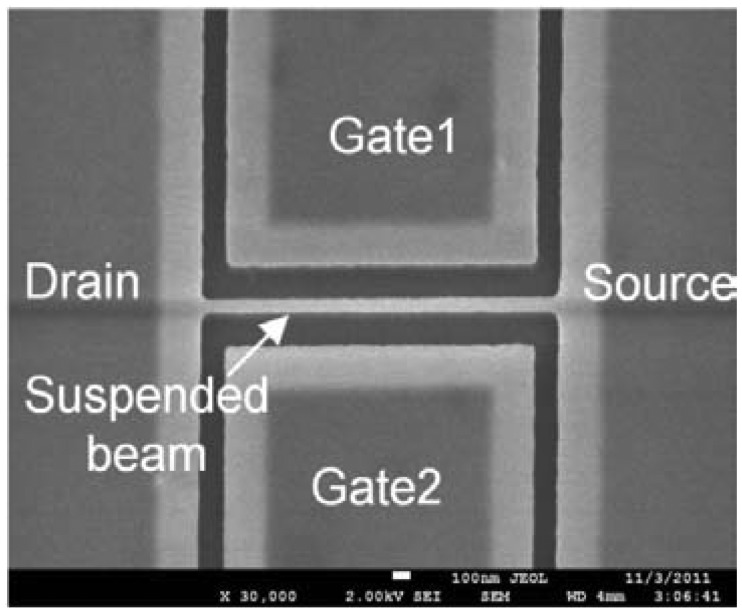
The scanning electron microscope (SEM) view of the fabricated in-plane resonant nano-electro-mechanical (IP R-NEM) sensor.

**Figure 10.**
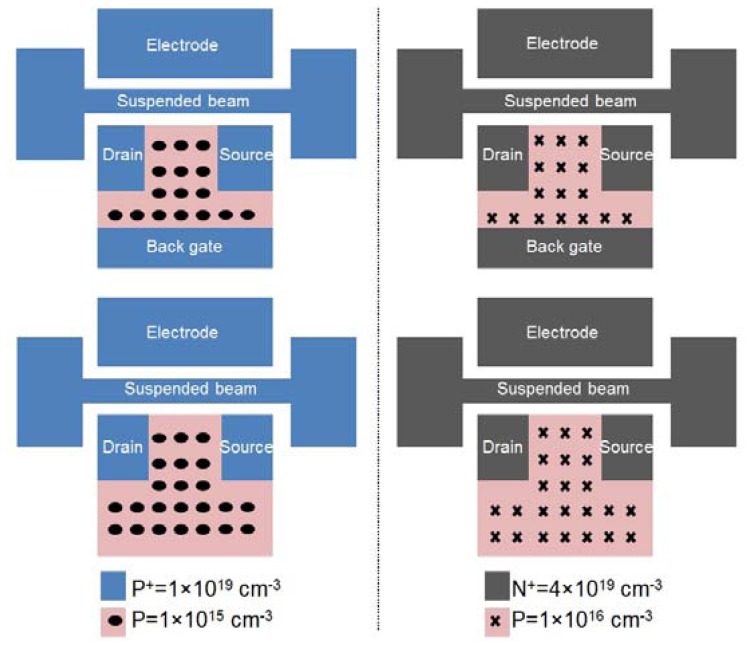
The doping strategies for the in-plane resonant suspended gate field-effect-transistor (IP RSG-FET) sensor: (left) P^+^/P/P^+^-type and (right) N^+^/P/N^+^-type metal-oxide-semiconductor field-effect-transistor (MOSFET) with/without the back gate on silicon-on-insulator (SOI).

**Figure 11.**
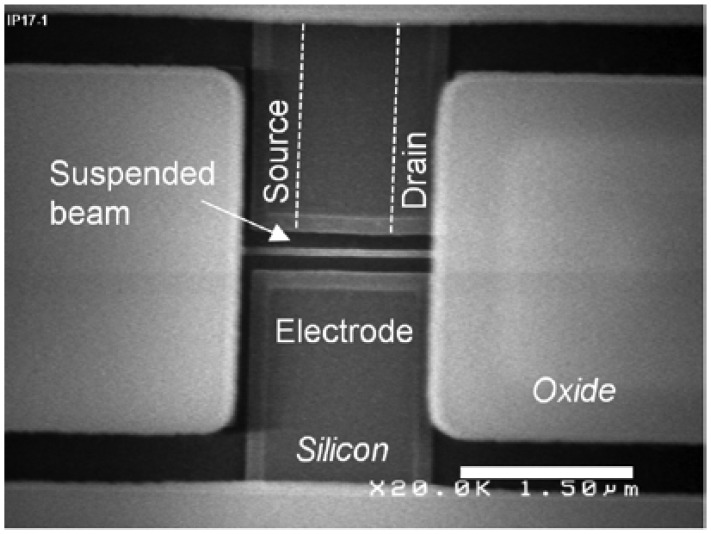
The scanning electron microscope (SEM) view of the fabricated in-plane resonant suspended gate field-effect-transistor (IP RSG-FET) sensor.

The IP R-NEM sensor was fabricated on an SOI platform. The thickness of the SOI and buried oxide (BOX) layers for this sensor are 40 nm and 145 nm, respectively. P-type doping of P = 10^15^ cm^−3^ is considered for the SOI wafer. First, the SOI wafer was implanted with the doping of P^+^ = 10^19^ cm^−3^. Then the heavily-doped silicon was patterned and the beam was released using vapor hydrofluoric (HF). A 15 nm-layer of thermal oxide was grown on the surface of the patterned silicon, especially around the suspended beam for passivation and minimizing of the silicon surface states [34]. After that, poly silicon was deposited to fill the etched area around the beam to protect the beam from further processing steps. Contact holes above the silicon pads are opened by etching poly silicon with the etch stop layer of thermal oxide. Then, oxide is deposited and contact holes are patterned in this layer. Aluminium (Al) is deposited and patterned for contact pads and wiring afterwards. Finally, a window is opened in oxide and poly silicon above the beam for the suspension of the beam using xenon difluoride (XeF_2_). The scanning electron microscope (SEM) image of the sensor is shown in Figure 9. The same fabrication steps of the IP R-NEM sensor applied for the IP RSG-FET sensor except considering two values of p-type doping, P = 10^15^ cm^−3^ and P = 10^16^ cm^−3^, for the SOI wafers. Moreover, during the implantation steps with different dopings of P^+^ = 10^19^ cm^−3^ and N^+^ = 4 × 10^19^ cm^−3^, the channels of the MOSFETs were protected by resist. The top view schematics in Figure 10 show the doping strategies for IP RSG-FET sensors. The SEM view of the sensor is shown in Figure 11.

## 5. Direct Current (DC) and High Frequency Characterization of Nano-Electro-Mechanical (NEM) Sensors

The detection of resonance frequency is the bottleneck of nano-electro-mechanical (NEM) resonators due to the need of good signal to noise/background ratio (SNR/SBR) to single out very small output signals [35]. For this reason, several high frequency techniques were applied for the NEM sensors to investigate the best resonance frequency detection method for the sensors with the current designs.

### 5.1. Characterization of In-Plane Resonant Nano-Electro-Mechanical (IP R-NEM) Sensor

**Figure 12.**
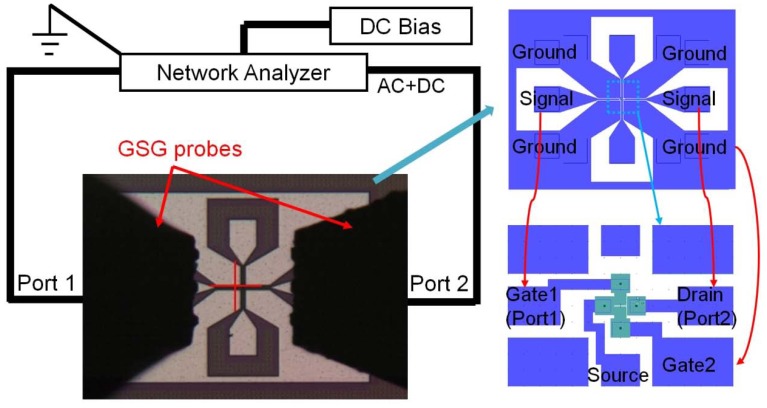
The radio frequency (RF) measurement setup for the in-plane resonant nano-electro-mechanical (IP R-NEM) sensor.

For the radio frequency (RF) characterization of the IP R-NEM sensor, Cascade SUMMIT 12000B probe station was used. The S-parameters for the sensor were measured using an Agilent E8361A PNA network analyzer. Ground-signal-ground (GSG) probes with the pitch of 150 μm were used for the measurement. The characterization setup is shown in Figure 12. The two-port calibration was done at the end of GSG probes. The RF characterization was done for the beam with *w* = 135 nm, *g* = 80 nm and *l* = 2,000 nm that has a resonance frequency of 285 MHz based on the numerical analysis. The AC voltage with the power of −10 dBmWatt, IF bandwidth of 500 Hz and number of point of 601 were applied. The DC voltage is applied to the beam using the Agilent semiconductor device analyzer B1500. The measurement was done in the atmosphere and at room temperature.

It was not easy to distinguish the resonance peak in the transmission signal, *S_21_*, from the background noise signal for this device. In order to explain this effect, the IP R-NEM sensor with the current dimensions is compared to the 14-MHz in-plane NEM resonator by Durand *et al.* [36]. Their resonator consists of a vibrating gate and a resonant suspended gate MOSFET (RSG-MOSFET) that is fabricated using silicon-on-nothing (SON) technology. The vibrating gate has the dimensions of *w* = 165 nm, *t* = 400 nm, *g* = 120 nm and *l* = 10 μm and measured parameters for the RSG-MOSFET resonator are: *f_0_* = 14.43 MHz, *R_x_* = 736 kΩ and *Q_Total_* = 700. The electrical setup for the capacitive detection of the RSG-MOSFET resonator is similar to the setup for the IP R-NEM sensor in Figure 12 and it showed 2 dB-magnitude peak for the transmission signal. The *R_x_* for the IP R-NEM sensor that works in the atmosphere is 100 times higher than that of the RSG-MOSFET using Equation (16). Due to this fact, the IP R-NEM sensor shows higher signal drop across the resistance and consequently smaller output signal than the 2 dB-magnitude transmission signal of the capacitive detection for the RSG-MOSFET. This reason explains the difficulty in distinguishing the resonance peak with very small magnitude from the back ground noise signal.

**Figure 13.**
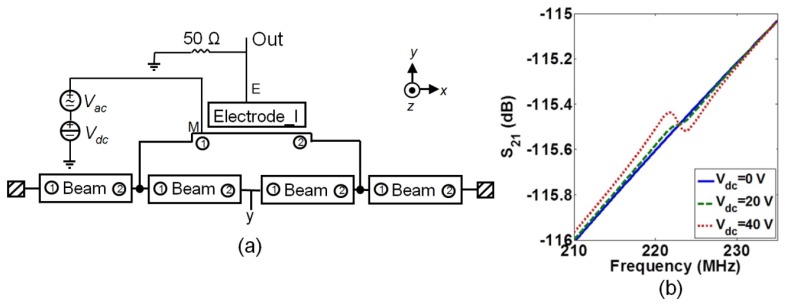
**(a)** The circuit model for the suspended beam with one side electrode using Architect for extracting *S_21_* signal and (**b**) the magnitude of the *S_21_* signal for different *V_dc_*.

The circuit-level simulation of an IP R-NEM sensor with one side electrode in Figure 13a was done to confirm the previous comparison between the IP R-NEM sensor and RSG-MOSFET. Figure 13b shows the very small magnitude of *S_21_* in the presence of different applied *V_dc_* that is consistent with our previous explanation. In order to reduce *R_x_* and improve the transmission signal for the sensor, the measurement should be done in high vacuum, same as the RSG-MOSFET. It is important to note, that the lower working pressure reduces *R_x_* if *Q_Total_* is mainly dominated by air damping, *Q_Ambient_*. For example, *Q_Total_* for the NEM sensor with *w* = 135 nm and *l* = 2,000 nm is dominated by anchor doping, *Q_Anchor_*, and its *R_x_* is not reduced much by using high vacuum. Use of different measurement techniques such as lock-in measurement [37–39], is a good option for the measurement of these devices.

**Figure 14.**
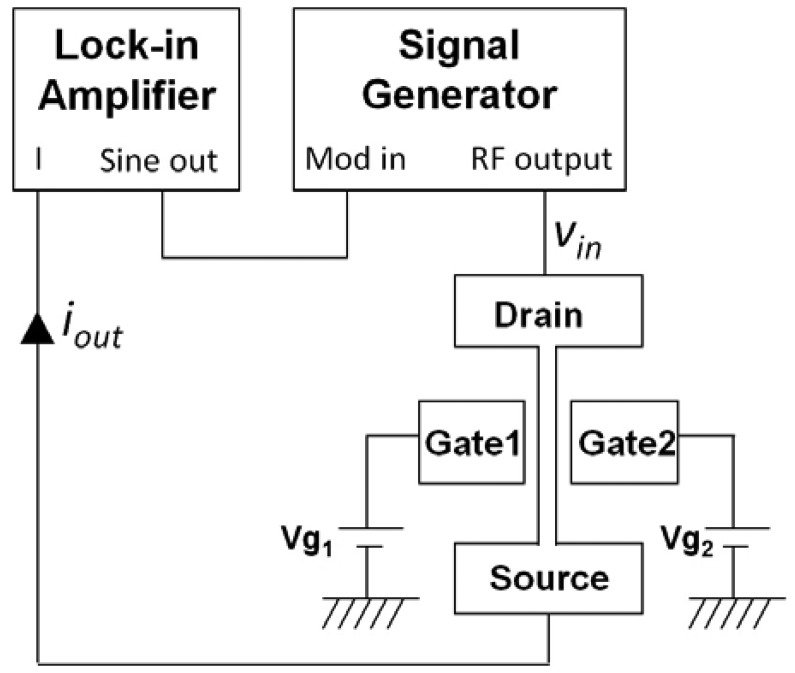
Down-mixing measurement setup for the in-plane resonant nano-electro-mechanical (IP R-NEM) sensor.

We have applied a down-mixing technique [40,41] for the RF characterization of the IP R-NEM sensor due to the above mentioned difficulties in the RF characterization of the sensor using a network analyzer. This current technique takes advantage of the intrinsic gain of the integrated JL transistor [42] within the IP R-NEM sensor. The measurement setup is shown in Figure 14.

By applying different voltage to gates, *V_g1_* and *V_g2_*, the strongest current modulation due to the movement of the beam will be achieved. The signal generator in Figure 14 was used to apply a frequency modulation carrier signal, *v_in_*, to drain. The measurements were done at high vacuum (10^−6^ mbar) and room temperature. The lock-in amplifier was used to detect the output current signal, *i_out_*, from source. The output current, *i_out_*, is defined by [40]:

(26) $$i_{\text{out}}\propto g_{DS}v_{in}\frac{\partial i_{DS}}{\partial y}y(\omega )$$

where *g_DS_* is the output conductance, *y*(*ω*) is the frequency, *ω*, dependent in-plane displacement. The changes in the drain current, *i_DS_*, to the displacement, *∂i_DS_*/*∂y*, is calculated by [43]:

(27) $$\frac{\partial i_{DS}}{\partial y}\approx g_{m}\frac{C_{eq}^{\prime }}{C_{eq}}V_{g}$$

where *g_m_* is the trransconductance, *C_eq_*, is the equivalent gate capacitance, *C′_eq_* is the derivative of *C_eq_*.

**Figure 15.**
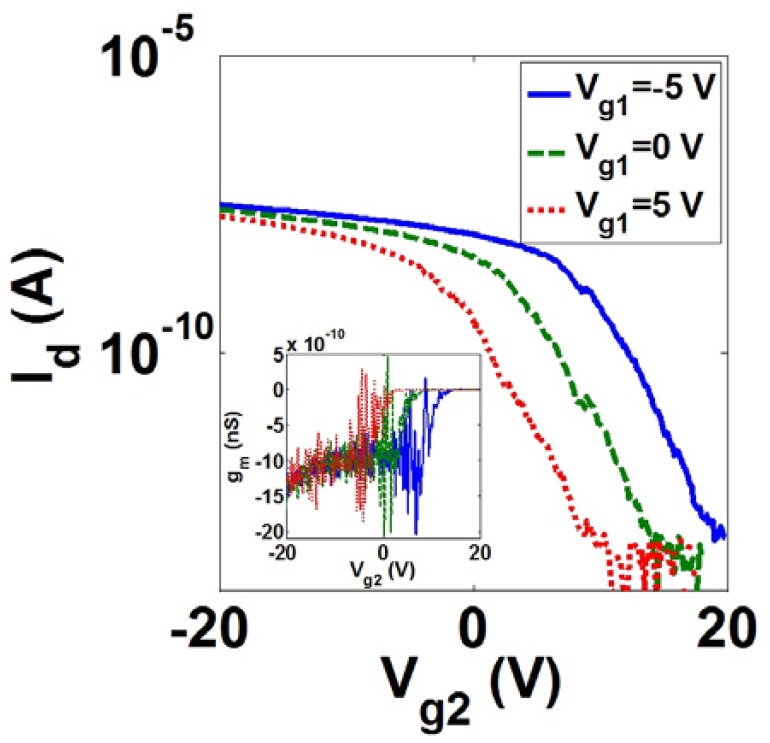
The effect of various *V_g1_* on direct current (DC) characteristics of the 1.5 μm-length in-plane resonant nano-electro-mechanical (IP R-NEM) sensor at *V_d_* = 50 mV.

Figure 15 shows the impact of applying different voltages to gates of the sensor *l* = 1.5 μm, *w* = 45 nm and *t* = 40 nm on its *I_d_*-*V_g_* and *g_m_*-*V_g_* (inset of Figure 15) characteristics. The asymmetrical applied voltages of *V_g1_* = −20 V and *V_g2_* = −5 V provides higher ON-current as well as high *g_m_*.

**Figure 16.**
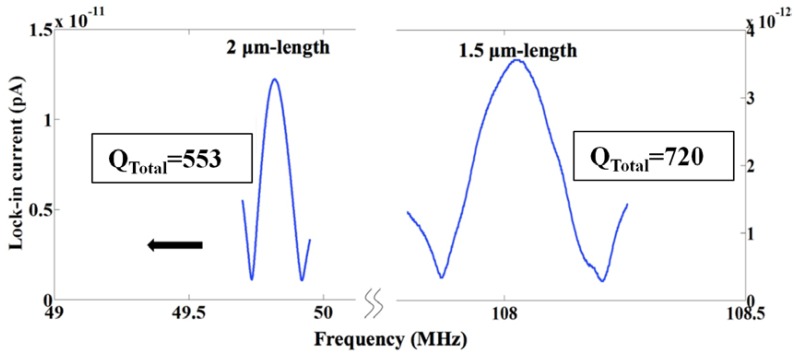
High frequency characteristics of in-plane resonant nano-electro-mechanical (IP R-NEM) sensors with different lengths of the suspended beam.

**Figure 17.**
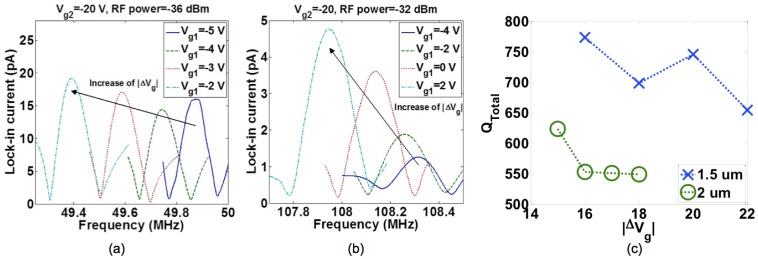
The impact of changing *V_g1_* on the resonance frequency of: (**a**) 2 μm-length and (**b**) 1.5 μm-length in-plane resonant nano-electro-mechanical (IP R-NEM) sensors. (**c**) *Q_Total_versus* |*ΔV_g_*| for IP R-NEM sensors with different lengths of the suspended beam.

**Figure 18.**
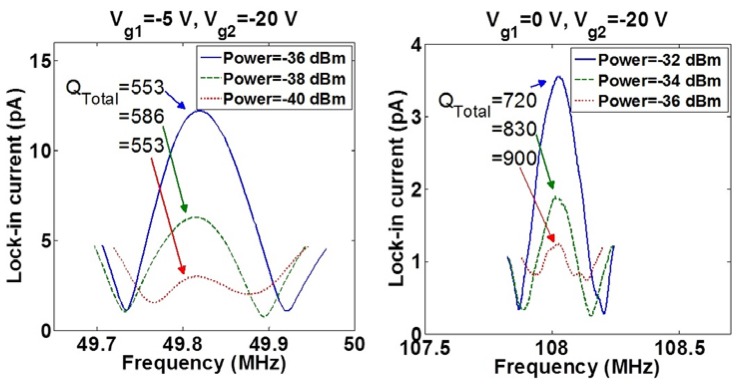
The effect of various radio frequency (RF) power on the frequency characteristics of the: **(left)** 2 μm-length and **(right)** 1.5 μm-length in-plane resonant nano-electro-mechanical (IP R-NEM) sensors.

The lock-in current *versus* frequency of two sensors with *l* = 1.5 and 2 μm are shown in Figure 16. The measured *f_0_* and *Q_Total_* are less than the analytical values of *f_0_* = 158.98 MHz and *Q_Total_* = 9,762 for *l* = 1.5 μm and *f_0_* = 89.42 MHz and *Q_Total_* = 23,141 for *l* = 2 μm considering a 15 nm-silicon dioxide (SiO_2_) layer around the beam. These differences can be explained due to changes of dimensions of the suspended beam from the original designed values. In order to investigate the impact of gates' bias voltages on the resonance frequency, *V_g2_* was fixed to −20 V while *V_g1_* was changed. The lock-in current of both sensors *versus* frequency characteristics by changing *V_g1_* are shown in Figure 17a,b. In both devices, an increase in |*ΔV_g_* = *V_g2_* − *V_g1_*| causes an increase in the electrical spring stiffness and reduces the total spring stiffness, which leads to the reduction of the resonance frequency so called softening effect. The *Q_Total_* of both sensors *versus* |*ΔV_g_*| are shown in Figure 17c. The reduction of *Q_Total_* by increasing |*ΔV_g_*| can be seen for both sensors especially for the shorter length beam due to the higher applied voltages. The lock-in current *versus* the frequency of resonators for various RF powers are shown in Figure 18. This figure shows the stability of the resonance frequency of sensors by increasing the RF power. However, the 2 μm length sensor shows the reduction of *Q_Total_* by increasing the RF power. This is due to the fact that by increasing the power, the temperature of the beam and consequently *Q_Thermoelastic_* will increase. Higher value for *Q_Thermoelastic_* leads to the dominancy of this factor in the total quality factor and the reduction of *Q_Total_* with respect to Equation (7). The dependence of the resonance frequency of the resonator to the value of applied RF power and |*ΔV_g_*| shows the necessity of doing the measurement at low temperatures. The reason for the increasing temperature in the beam may be explained by the existence of the thermal oxide layer around the beam. This is due to the fact that oxide has smaller thermal conductivity in comparison with silicon, which results in less dissipation of heat to the environment. For this reason, the fabrication of a suspended beam without thermal oxide will improve this temperature dependency. Applying the same measurement technique for the functionalized sensor will be done in the future.

### 5.2. Characterization of In-plane Resonant Suspended Gate Field-Effect-Transistor (IP RSG-FET) Sensor

**Figure 19.**
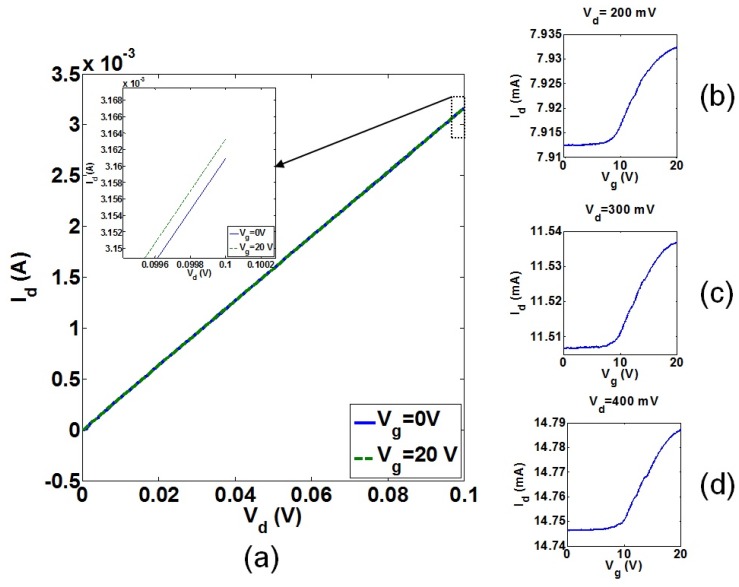
The N^+^/P/N^+^-type in-plane metal-oxide-semiconductor field-effect-transistor (MOSFET) with *w* = 135 nm, *g* = 80 nm and *l* = 2,000 nm: (**a**) the *I_d_*-*V_d_* characteristic at *V_g_* = 0 and 20 V and *I_d_*-*V_g_* characteristic at (**b**) *V_d_* = 200 mV, (**c**) *V_d_* = 300 mV, (**d**) *V_d_* = 400 mV.

As discussed before, a MOSFET is integrated with the beam for the IP RSG-FET sensor to improve the magnitude of the transmission signal in which the output signal from the NEM structure is amplified by the intrinsic gain of MOSFET [26,36], *g_m_* × *r_o_*, where *r_o_* is the output resistance of MOSFET. Similar to JL FET detection method for the IP R-NEM sensor, by optimizing the applied bias voltages in DC characteristics of the MOSFET, the maximum value for *g_m_* is achieved that amplifies the output signal of the MOSFET as much as possible. Based on the fabrication technology, after the implantation and drive-in steps for dopants, there is an estimated lateral diffusion length of 100 nm. The voltage of 0–0.1 V was applied to drain, *V_d_*, for the enhancement-mode n-channel MOSFET (N^+^/P/N^+^-type) with channel length, *l_C_*, of 1,250 nm, *l* = 2,000 nm, *w* = 135 nm and *g* = 80 nm. The drain current-drain voltage, *I_d_*-*V_d_*, characteristics of the MOSFET with different applied voltages to the suspended beam, *V_g_* = 0 and 20 V, are shown in Figure 19a. Figure 19a shows the very small control of gate over the channel. The drain current-gate voltage, *I_d_*-*V_g_*, characteristics of the MOSFET is shown in Figure 19b–d for *V_d_* in the range of 200 to 400 mV. Figure 19b–d show the OFF-current in the order of raA and a very small ON/OFF current ratio. The current in Figure 19b–d increases by increasing *V_d_*, which shows the strong impact of drain over the channel. The threshold voltage, *V_t_*, of 9 V in Figure 19b–d is much larger than the analytically calculated value of *V_t_* = 1.75 V for the conventional long n-channel MOSFET. This difference in the threshold voltages cannot be explained due to the drain-induced-barrier-lowering (DIBL) effect which should cause the reduction in the threshold voltage [42]. The same order of OFF-current has been found also for the depletion-mode p-channel MOSFET (P^+^/P/P^+^-type) with the same dimensions of the n-channel MOSFET.

In order to investigate the origin of high OFF-current, the leakage of the n-channel MOSFET from source and drain to the back gate was measured. The leakage current was in the order of 10^−4^ A for both drain and source and 1 μm-distance of back gate from source and drain shows the dopants diffusion of source and drain towards the back gate is more than the previously stated 100 nm. The value of diffusion length that gives this level of leakage current was found by simulating the device in ATLAS 3D [44]. The simulation results showed that by considering the dopant diffusion of 400 nm and a negative charge of −1 × 10^12^ cm^−3^ in the interface of the oxide layer and silicon beam, the same *I_d_*-*V_g_* characteristics for the MOSFET in Figure 19 will be achieved. In order to isolate the source and drain as much as possible, a trench is milled between them using focused ion beam (FIB), which improved the *I_d_*-*V_g_* characteristics negligibly. Designing of MOSFETs with a longer channel length may be a solution to avoid the issue of high OFF-current in these sensors.

## 6. Conclusions

In this paper we have presented the design, simulation, fabrication and characterization of in-plane resonant nano-electro-mechanical (NEM) sensors. These sensors are based on the mass detection principle and can be used as a bio/chemical sensor. The proposed sensors were designed and simulated using both three dimensional finite-element-method (3D FEM) simulation and hybrid nano-electro-mechanical metal-oxide-semiconductor (NEM-MOS) circuit simulation. The surface of the suspended beam should be functionalized for adsorption of target molecules. The linker and target molecules of the sensing process have been modeled by adding extra layers to the beam in different configurations for investigating the extreme mass responsivity of 0.05 zepto g/Hz for the sensors. The in-plane resonant nano-electro-mechanical (IP R-NEM) and in-plane resonant suspended gate field-effect-transistor (IP RSG-FET) sensors were fabricated successfully. The radio frequency (RF) characterization of the IP R-NEM senor was investigated in different analytical and numerical levels to clarify the best characterization method for the sensors with current specifications. Down-mixing technique was successfully applied for the RF characterization of the IP R-NEM sensors as the suitable high frequency characterization technique. Due to the challenges of the newly proposed fabrication technology of the IP RSG-FET senor, some post processing and simulations were done to investigate and improve the direct current (DC) characteristic of this sensor. The numerical analysis shows the impact of the lateral diffusion of source/drain dopants and also the possible trapped charges in the surface of the vertical channel of the MOSFET on the DC characteristics of the sensor. Further post-processing and design optimization should be conducted to improve or avoid the discussed challenges for IP RSG-FET sensors.

## Figures and Tables

**Table 1.** Parameters used for calculations and the derived analytical values for nano-electro-mechanical (NEM) sensors.

**Parameters Used for Calculations**			**Analytical Values**	

**Dimensions**	**Silicon Properties**	**Ambient Properties**	**Key Quantities**	**Quality Factors**
*t* = 100 nm	*E*= 130.18 GPa	*ε_0_* = 8.85 × 10^−12^ F/m	*f_0_* = 384.95 MHz	*Q_Ambient_* = 2,086
*w* = 50 nm	*p* = 2,331 kg/m^3^	*μ_0_* = 1.86 × 10^−5^ kg/(m.s)	*m_b_* = 8.56 fg	*Q_Thermoelastic_* = 868,352
*l* = 1 μm	*α*′ = 2.616 × 10^−6^ 1/K	*μ* = 1.259 × 10^−6^ kg/(m.s)	*k_bm_* = 50.08 N/m	*Q_Anchor_* = 4,998
*g* = 50 nm	*C* = 700 J/(kg.K)	*P_n_* = *P_0_* = *P_atm_* = 101,325 Pa	*V_p_* = 45.41 V	*Q_Total_* = 1,469
	*χ* = 0.86 cm^2^/s	*λ_n_* = *λ_0_* = *λ_atm_* = 68 nm	*b* = 9.926 × 10^−12^ (N.s)/m	
	*ϑ* = 0.33	*T_0_* = 300 K		
		**Other Parameters**		
		*β_0_* = 1.5056, *X_0_* = −0.983		

**Table 2.** The derived numerical values for nano-electro-mechanical (NEM) sensors.

**Numerical Values**	

**Key Quantities**	**Quality Factors**
*f_0_* = 432.47 MHz *b* = 9.075 × 10^−12^ (N.s)/m *V_p_* = 56.95 V	*Q_Ambient_* = 2,563 *Q_Thermoelastic_* = 680,633 *Q_Anchor_* = 3,154 *Q_Total_* = 1,411

**Table 3.** The effect of the increasing of the thickness of the coating layer in different configurations on the resonance frequency.

**Configuration**	***Δm****_bl_***/*m****_b_*	***Δk****_bl_***/*k****_b_*	**Dominant Parameter**	***Δf****_0_*
OT	0.81%	0.51%	*m_b_*	<0
TB	1.75%	1.07%	*m_b_*	<0
AA	5.60%	7.90%	*k_b_*	>0

**Table 4.** Comparison of the in-plane resonant nano-electro-mechanical (IP R-NEM) sensor and a few recent mass detection based NEM sensors.

**Reference**	**Material**	**Frequency (Hz)**	***Q****_Total_*	**Temperature**	**Medium**	**Mass Responsivity or Detection Limit**
[12]	SiC	32.8 M	3,000	17 K	Ultra High Vacuum	2.53 atto g
[30]	SiC	428 M	2,500	22 K	Vacuum	0.27 zepto g/Hz
[4]	SiC	190 M	5,000	300 K	Ultra High Vacuum (<10^-10^ Torr)	0.86 zepto g/Hz
[32]	Carbon nano-tube (Fe-coated)	470 M	15	300 K	Vacuum (10^−6^ Torr)	∼1 atto g
[33]	Silicon nano-wire (Metallized)	200 M	2,000	25 K	Vacuum	0.71 zepto g/Hz
Nano-electro-mechanical (NEM) sensors in this paper	Silicon	*Analytical*		300 K	Atmosphere	*Analytical*
		384.95 M	1469			0.007 zepto g/Hz
		*Numerical*				*Numerical*
		432.47 M	1411			0.05 zepto g/Hz
